# The European hedgehog (*Erinaceus europaeus*), as a reservoir for helminth parasites in Iran

**Published:** 2015-06-15

**Authors:** Soraya Naem, Behzad Pourreza, Tahmineh Gorgani-Firouzjaee

**Affiliations:** 1 *Department of Pathobiology, Faculty of Veterinary Medicine, Urmia University, Urmia, Iran; *; 2 *Department of Surgery and Radiology, Faculty of Veterinary Medicine, Tehran University, Iran; *; 3 *Department of Pathobiology, Faculty of Veterinary Medicine, Shahid Bahonar University of Kerman, Kerman, Iran.*

**Keywords:** *Brachylaemus erinacei*, *Crenosoma striatum*, Hedgehog; Iran, *Physaloptera clausa*

## Abstract

From April 2009 to December 2011, 44 dead hedgehogs (*Erinaceus europaeus*) were collected incidentally from areas of Urmia, Iran. The overall prevalence of helminth infections was 95.0%. Specific parasites and their prevalences were: *Physaloptera* *clausa* (93.0%), *Crenosoma striatum *(61.0%)*, Capillaria* *aerophila* (9.0%)*, Capillaria*s spp. (4.0%), *Brachylaemus* *erinacei* (2.0%) and *Hymenolepis* *erinacei* (16.0%)*.* There were no significant differences in helminth occurrence between hedgehog sexes, either in single or in mixed infections (*p *> 0.05). The mixed infection involving *Crenosoma striatum* and *P. clausa* occurred significantly more frequently than other mixed infection (*p *< 0.05). There were significant differences in prevalence among seasons, with the highest prevalence in summer and spring especially among *P. clausa* and* C. striatum (p *< 0.05).

## Introduction

The European hedgehog (*Erinaceus europaeus*) is a common and well-known species, native to Europe, including European Russia, and Iran.^1^ It has been introduced to several European islands and as an exotic species to New Zealand. The European hedgehog is omnivorous, however, feeds mainly on invertebrates, including slugs, earthworms, beetles, caterpillars, and other insects. It also eats grass snakes, vipers, frogs, fish, small rodents, young birds, and birds' eggs. Because of its dietary habits, the hedgehog may act as a definitive or paratenic host of agents that pose a risk for some zoonotic diseases such as *Trichinella*.^2^^,^^3^ One nematode found in the stomach of hedgehogs, *Phyasoloptera clausa*, may is a vector for human leptospirosis.^4^There are reports of parasitic helminths of hedgehogs in several countries.^5^^-^^12^ For these reasons, we conducted the first evaluation of the prevalence and intensity of parasitic helminths in hedgehogs in Urmia, northwest of Iran.

## Materials and Methods

**Sample collection. **From April 2009 to December 2011, 44 carcasses of European hedgehogs (21 females and 23 males) were collected from different parts of Urmia including urban, suburban, garden areas and referred to the Department of Pathobiology, Faculty of Veterinary Medicine, Urmia University, Iran. Some hedgehogs were found dead on farms and others had been killed by motor vehicles on roads. The number of hedgehogs among in spring, summer and autumn was 22, 7 and 15, respectively. Urmia is located in northwest of Iran (45°2΄W, 37°32΄N), and has a semi-humid climate, and annual precipitation is approximately 350 mm with a mean maximum temperature of 28.3 ˚C in August and mean minimum temperature of – 5 ˚C in January.^13^

**Necropsy. **After collecting the animals, data about weight and sex were recorded. Hedgehogs were classified according to Pfaeffle: hoglets (< 100g), juveniles (< 500g), and adults (> 500g) with the number of 2, 12, and 30, respectively.^14^ Then, a routine necropsy was performed. The organs of the thoracic cavity, trachea, lungs, and heart, were dissected. After washing, the lungs were assessed visually for nematodes and examined using a stereo microscope to find small worms in the lung parenchyma. Worms were removed using forceps and kept in alcohol-formalin-acetic acid (AFA) solution (including 40.0% ethanol, 6.0% formaldehyde, 4.0% acetic acid, 45.0% distilled water, 5.0% glycerine).^15^ The same procedure was used for examining the heart, liver, spleen, and kidneys. The esophagus, stomach, and intestines were opened in separate trays using scissors, washed, and mucosa carefully examined. The contents of stomach and intestines were examined using a stereo microscope. All nematodes were fixed in AFA, cleared with lactophenol solution (25.0% glycerine, 25.0% lactic acid, 25.0% phenol and 25.0% distilled water) and examined using a light microscope. Cestodes and trematodes were removed from the small intestine, washed, and kept in cool water for two days. The routine technique was applied to staining and mounting of plathyhelminths as described by Soulsby.^15^ Identification of species was confirmed under light microscopy using the keys of Yamaguti and Beck.^10^^,^^16^

**Statistical analysis. **Prevalence (%), mean intensity, and mean abundance were evaluated following Bush *et al*.^17^ Chi-square and Fisher’s exact tests were used to test significance of differences in helminth infection prevalence using SPSS (Version 16; SPSS Inc., Chicago, USA). Differences were considered significant at *p *< 0.05.

## Results

**Fig. 1:**
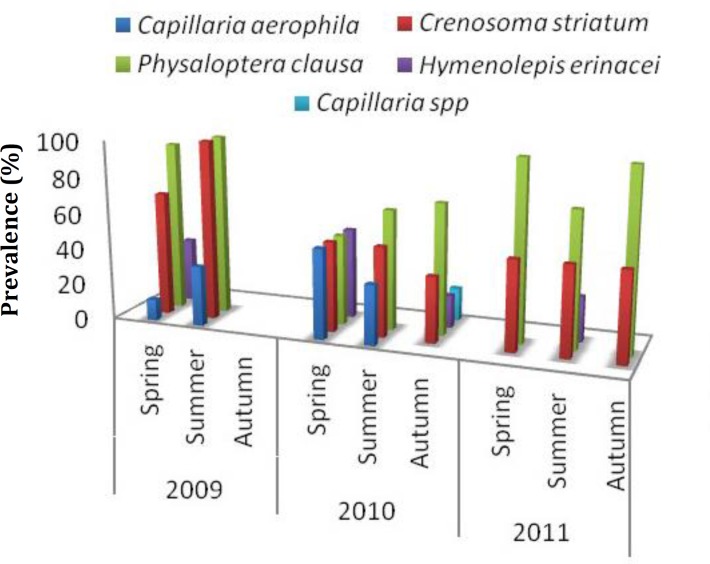
Seasonal prevalence of helminth infection by species in 44 hedgehogs (*Erinaceus europaeus*), April 2009 – December 2011, in Urmia, Iran. Spring = April – June; Summer = July – September; Autumn = October – December

Forty-two of 44 hedgehogs (95.0%) were infected with helminths. Four nematodes (*Crenosoma striatum, Physaloptera clausa*, *Capillaria aerophila*, and *Capillaria *spp.), one trematode (*Brachylaemus erinacei*), and one cestode (*Hymenolepis erinacei*) were isolated (Table 1).The highest numbers of helminths were isolated in the stomach and the fewest in the small intestine. There were no statistically significant differences in helminthic infection prevalence between hedgehog sexes, either for single or mixed infections. There was no infection in hoglets. Infection rates in juvenile and adult group were 100%. The intensity of infection was high in adults. Of all animals were examined and 32.0% were infected with only one species. *Physalopptera clausa *and *C. striatum *were the most frequent mixed infection (43.0% of hedgehogs) and this pairing occurred significantly more frequently than other mixed infections (*p *< 0.05). In one case several cysts filled with numerous individuals of *P. clausa *were found on the surface of spleen, liver, and uterus. The male and female worms were 22 to 30 and 28 to 47 mm long, respectively. The white, stout worms had the cuticle reflected over the lips to form a large cephalic collarette. *Crenosoma striatum *was also common among hedgehogs. The males and females were white, and 10-15 and 15-20 mm long, respectively. In 2009, all hedgehogs (17), were infected with the prevalence of 100% in spring and summer. No infection was found in autumn. In year two, all 15 examined animal, were infected and parasite burdens were commonly distributed among the seasons. In 2011, 95.0% of animals (12) found infected. Highest prevalence of infection was in summer and spring, especially with *C. striatum* and *P. clausa *in three years of study which had significant differences with other helminths (Fig. 1). *Capillaria aerophila *showed a low overall prevalence (9.0%).

**Table 1:** Prevalence and intensity of helminth species in 44 hedgehogs (*Erinaceus europaeus*) from Urmia, Iran

**Species (Site of infection)**	**No. positive (Prevalence[%])**	**Mean intensity (± SD)**	**Mean abundance (± SD)**	**Range**
***Crenosoma striatum *** **(** **Lungs)** ***Capillaria aerophila *** **(** **Lungs)** ***Physaloptera clausa *** **(** **Stomach)** ***Capillaria *** **spp** **. (Small intestine)** ***Hymenolepis erinacei *** **(** **Small intestine)** ***Brachylaemus erinacei *** **(** **Small intestine)**	27(61) 4(9) 41(93) 2(5) 7(16) 1(2)	20.5 (± 15.5) 25.4(± 29.1) 25.6(± 16.2) 0.2 (± 1.4) 3.6(± 1.4) 1	19.1 (± 16.0) 25.6(±16.2) 28.2 (± 30.7) 6(± 4.2) 0.6(± 1.4) 0.2(± 0.1)	1-65 1-122 1-108 1-9 1-5 1

## Discussion

Morphologic details of *P. clausa*, the most commonly observed nematode, have been reported by Gorgani *et al*. using scanning electron microscopy.^18^ Adult nematodes of the genus *Physaloptera* occur mainly in the stomach, and seldom in the small intestine of amphibians, reptiles, birds, and a wide range of insectivorous and omnivorous mammals throughout the world.^19^ This nematode has an indirect life cycle and insects including beetles, cockroaches, and cricketsare intermediate hosts; reptiles are paratenic hosts.^20^^,^^21^ The preferred arthropods are the millipedes,* Glomerismarginata* and *Tachypodoiulusniger*, and the ground beetle, *Carabus nemoralis*.^22^ Hedgehogs also eat grass snakes, vipers, frogs, fish, small rodents, young birds and birds' eggs and some fruits and mushrooms may supplement the diet. Because the larval stages of the parasites occur in all of these hosts, this kind of diet facilitates completing the life cycles of parasitic helminths including* P. clausa*,* C. striatum*,* B. erinacei* and* H. erinaceus*, which can infect the European hedgehog.

*Physaloptera clausa *firmly attach to the wall of stomach and duodenum, where they feed on mucosa and suck blood.^15^*Physaloptera *species and particularly *P*. *clausa*has been associated with severe gastric lesions in Israel, Japan, Canada, USA, Italy, Germany, Poland, Turkey, and Iran.^4^^,^^9^^,^^10^^,^^24^^-^^31^

Diagnosis of *Physaloptera* species infection is made based on clinical signs and the presence of parasite eggs in the feces.^23^ In a study which was carried out on eastern European hedgehog (*Erinaceus concolor*) in Mazandaran province, Iran, infection with *P. clausa *(40.0%) was more prominent than other parasites.^12^ Cirak *et al*. studying helminth parasites of hedgehogs in Turkey, also found that* P. clausa* was the most common nematode (72.2%), findings similar to the present study.^30^ Conversely, only 13.6% of examined hedgehogs in Poland were infected with *P. clausa*.^11^ Possible factors in the low prevalence in Poland might be climatic conditions and the scarcity of insects in the diet.

Another nematode with a high infection rate was *C. striatum,* a common infestation among hedgehogs. The worms are ovoviviparous and the life cycle is indirect. *C. striatum *uses snails and slugs as intermediate hosts, which are a common food source for European hedgehogs while *C. aerophila *is transmitted directly or indirectly by earthworms.^10^ *Crenosoma striatum *is associated with respiratory signs and, together with *Bordetella bronchi-septica,* has been associated with death of hedgehogs.^23^ Naem *et al*. studied pathologic lesions of hedgehog lungs infected by *C. striatum.*^27^ Majeed *et al*. found 14.0% of hedgehogs from England infected with *C. striatum*, 5.0% with *C. aerophila,* and 16.0% had amixed infection.^7^ The authors suggested that heavy infections of both species may cause severe inflammatory reaction or even death. *Capillaria aerophila*, a causative agent of pulmonary disease in carnivores, has great zoonotic importance and human infection is reported from Iran.^21^^-^^33^

The prevalence of *C. striatum *and *C. erinacei *in hedgehogs from England was 77.0 % and 85.0 %, respectively.^34^ Several coprologic studies of hedgehogs revealed that *C. striatum *and *C. aerophila *had the highest prevalence.^5^^,^^6^^,^^35^ We also detected the parasitic helminths *B. erinacei *in one hedgehog (2.0%). Infection with *B. erinacei *in hedgehogs can lead to restlessness, strong appetite with an excessive loss of weight at the same time, diarrhea with blood in the stool, hemorrhagic enteritis, and inflammation of the bile ducts with secondary bacterial infections, anemia, and even death.^10^ Krehmer described a case of a juvenile hedgehog infected with 2,516 trematodes which lead to its death.^36^ There have been many reports of infection with *B. erinacei *in parts of Germany.^5^^,^^37^ In one German study, 216 (93.1%) of 232 investigated hedgehog gastrointestinal tracts were parasitized with *B. erinacei, *and *Capillaria *sp.^6^ Infections with cestodes in hedgehogs are not common, however, when they occur, they normally involves *Hymenolepis erinaceus. *Clinical signs are diarrhea and weight loss.^10^ Prevalence of cestodes is normally very low. Timme described prevalence of 3.2% in Germany;^37^ Barutzki *et al*. found prevalence from 0 to 3.7% in Bavaria.^5^ Boag and Fowler reported 8.0% in northeast Scotland and in Cumbria, northern England.^33^ In the present study, 16.0% of examined hedgehogs were infected with this tapeworm.

We found the highest prevalence of helminth infection in spring and summer during the three years of the study. In the third year, *P.clausa *and *C.striatum* showed the highest prevalence especially in autumn. No infection was observed in winter because of hibernation in hedgehogs’ seasonal cycle. Our patterns were consistent with Dziemian *et al*. Although these authors studied ticks, they found a similar seasonal pattern in ectoparasite prevalence.^38^

In this study we observed mixed infections with *P. Clausa *and *C. Striatum *being most abundant. In another study in Turkey, prevalence of *P. clausa *(72.2%), *C. striatum *(55.5%), and *H. erinacei* (55.5%), were in agreement with our study.^29^ In an investigation of hedgehogs in several parts of Europe, the highest prevalence was associated with *Capillaria*s spp. (both lung and intestinal species), and *C. striatum*.^11^ There were no significant differences between male and female hedgehogs in the prevalence of infection by individual parasite species, either for single or in mixed infections. In a study carried out by Gaglio *et al*. in Britain, parasitic helminths removed from examined hedgehogs included *C. striatum *(70.0% of hedgehogs), *Capillaria *spp*. *(66.0% of stomach and 61.0% of intestine), and *B. erinacei *(55.0% of hedgehogs).^39^ We observed no significant differences in the abundance of any helminths between hedgehog sexes, or among seasons. The authors suggested that the worm burden was positively associated with body condition. Generally, parasite prevalence was related to age of the host. However, they used a mass: length regression as a body condition index for explaining prevalence.^39^ Because we did not have data on body length, we classified hedgehogs according to the weight classification of Pfaeffle: hoglets (< 100g), juveniles (< 500g), and adults (> 500g).^14^ The intensity of infection was significant in adults (*p* < 0.05). The reason may be related to growth of the animal Increasing weight gain may be accompanied by increasing use of dietary sources (i.e., mollusks and insects) that result in increased worm burden. Our results were consistent with the study by Majeed *et al*. who found higher lungworm burdens with increasing age and weight of British hedgehogs.^7^ Our findings concluded the high prevalence of zoonotic, parasitic helminth infections in hedgehogs in Urmia, northwest of Iran which underscores the importance of studies on hedgehog parasites in other areas of Iran.
